# Traceable Lactate-Fueled Self-Acting Photodynamic Therapy against Triple-Negative Breast Cancer

**DOI:** 10.34133/research.0277

**Published:** 2024-01-17

**Authors:** Yifan Zhang, Guangle Feng, Ting He, Min Yang, Jing Lin, Peng Huang

**Affiliations:** Marshall Laboratory of Biomedical Engineering, International Cancer Center, Shenzhen Key Laboratory of Tumor Visualization Molecular Medicine, Laboratory of Evolutionary Theranostics (LET), School of Biomedical Engineering, Shenzhen University Medical School, Shenzhen University, Shenzhen, 518055, China.

## Abstract

The depth of light penetration and tumor hypoxia restrict the efficacy of photodynamic therapy (PDT) in triple-negative breast cancer (TNBC), while the overproduction of lactate (LA) facilitates the development, aggressiveness, and therapy resistance of TNBC. To address these issues, a self-acting PDT nanosystem (HL@hMnO_2_-LOx@HA) is fabricated by loading 2-(1-hexyloxyethyl)-2-devinyl pyropheophorbide-alpha (HPPH), luminol, and LA oxidase (LOx) in a hyaluronic acid (HA)-coated hollow manganese dioxide (hMnO_2_) nanoparticle. LOx catalyzes the oxidation of LA into pyruvate and hydrogen peroxide (H_2_O_2_), thus depleting the overproduced intratumoral LA. In the acidic tumor microenvironment, H_2_O_2_ reacts with luminol and hMnO_2_ to yield blue luminescence as well as O_2_ and Mn^2+^, respectively. Mn^2+^ could further enhance this chemiluminescence. HPPH is then excited by the chemiluminescence through chemiluminescence resonance energy transfer for self-illuminated PDT. The generated O_2_ alleviates the hypoxia state of the TNBC tumor to produce sufficient ^1^O_2_ for self-oxygenation PDT. The Mn^2+^ performs *T*_1_ magnetic resonance imaging to trace the self-acting PDT process. This work provides a biocompatible strategy to conquer the limits of light penetration and tumor hypoxia on PDT against TNBC as well as LA overproduction.

## Introduction

Triple-negative breast cancer (TNBC) is a subtype of breast cancer with the poorest prognosis mainly because of the paucity of effective therapies other than systemic chemotherapy and its aggressive behavior [1,2]. Photodynamic therapy (PDT) enables targeted cancer therapy through spatiotemporally controlled light illumination and can eradicate tumor cells as sufficient singlet oxygen (^1^O_2_) is generated [3,4]. Moreover, PDT has several distinct advantages over conventional chemotherapy and radiotherapy including noninvasiveness and repeatability without cumulative toxicity [5]. Nevertheless, the hypoxic condition of TNBC tumors severely hampers an adequate supply of oxygen for PDT [6–8]. Moreover, the penetration depth of light in the TNBC tumor is limited because of the surface reflection, tissue scattering, tissue autofluorescence, and absorption by endogenous chromophoric biomolecules (e.g., heme groups, various forms of melanins, and aromatic amino acid residues in proteins) [9,10]. To avoid the tissue penetration process, direct activation of photosensitizers using a coadministered or coloaded energy source via chemi-/bioluminescence resonance energy transfer (CRET/BRET) [11–13], chemically initiated electron exchange luminescence [14], or Cherenkov radiation energy transfer [15] represents an intriguing avenue for effective PDT in deep solid tumors including TNBC [16]. Nevertheless, the efficacy of self-excited PDT in TNBC is still limited with the hypoxic tumor microenvironment. The research on oxygen supply for self-excited PDT is still in its fancy [17,18].

The overproduction of lactate (LA) [19,20] in TNBC tumors is associated with rapid progression, aggressiveness, and resistance to conventional chem/radio/immunotherapy [21–24]. Several approaches have been reported to modulate the reprogrammed LA metabolism in TNBC cells [25], including inhibition of LA dehydrogenase [26], monocarboxylate transporters [27,28], and the LA receptor GPR81 [29]. For example, Shao et al. [26] found that the LA dehydrogenase inhibitor FX-11 could effectively sensitize the 4T1 xenograft tumors to anti–programmed cell death protein 1 treatment and showed a markedly increased tumor infiltration of CD8^+^ T cells and natural killer cells. As a natural enzyme, LA oxidase (LOx) could consume intratumoral LA by catalyzing its oxidation into pyruvate [30,31]. Moreover, this reaction produces H_2_O_2_, which triggers the subsequent treatment modalities by chain reactions such as chemotherapy [32,33] and chemodynamic therapy [34,35]. However, it lacks feasible approaches to monitor the chain reactions in vivo.

**Fig. 1.**
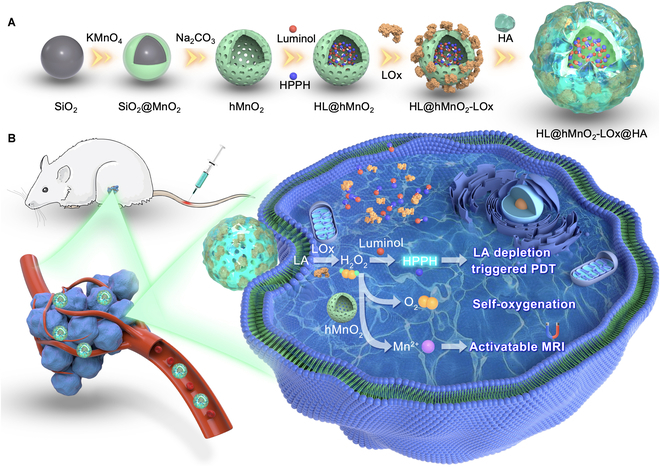
Schematic illustration of the FLI/MRI traceable LA-fueled self-acting PDT against TNBC. (A) Preparation procedure of the self-acting PDT nanosystem (HLMLH). (B) In the HLMLH nanosystem, the overproduced LA in TNBC tumors acted as the fuel and generated H_2_O_2_ under the catalysis of LOx. The generated H_2_O_2_ reacted with luminol and emitted blue luminescence to excite HPPH for self-illuminated PDT and reacted with hMnO_2_ to produce O_2_ for self-oxygenation PDT and Mn^2+^ for activatable MRI.

Herein, a traceable LA-fueled self-acting PDT nanosystem was fabricated by coloading 2-(1-hexyloxyethyl)-2-devinyl pyropheophorbide-alpha (HPPH or Photochlor), luminol, and LOx in a hollow manganese dioxide (hMnO_2_) nanoparticle. To enhance the tumor-targeting capability and protect the LOx from hydrolysis, the as-prepared nanoparticle was decorated with hyaluronic acid (HA) (HL@hMnO_2_-LOx@HA, HLMLH). HPPH is a second-generation photosensitizer already in Phase II human clinical trials [36]. Initially, LOx consumed the intratumoral LA and generated H_2_O_2_. Afterward, the produced H_2_O_2_ oxidized luminol to yield an aminophthalate ion and emitted blue luminescence which peaked at about 440 nm (Fig. 1) [11,37]. The chemiluminescence then activates HPPH via CRET between luminol and HPPH for fluorescence imaging (FLI)-guided PDT [38]. Simultaneously, the nanocarrier hMnO_2_ was degraded by H_2_O_2_ in the acidic tumor microenvironment and generated O_2_ to improve the hypoxia of TNBC tumors as the source of PDT. This process was accompanied by the generation of Mn^2+^, which not only acted as a *T*_1_ contrast agent for activatable magnetic resonance imaging (MRI) [39] but also catalyzed the decomposition of H_2_O_2_ to produce •OH and enhanced the chemiluminescence [40]. As a result, the HLMLH nanosystem was expected to perform MRI/FLI traceable self-illuminated/-oxygenated PDT against TNBC.

## Results

### Preparation and characterizations of the self-acting PDT system

**Fig. 2.**
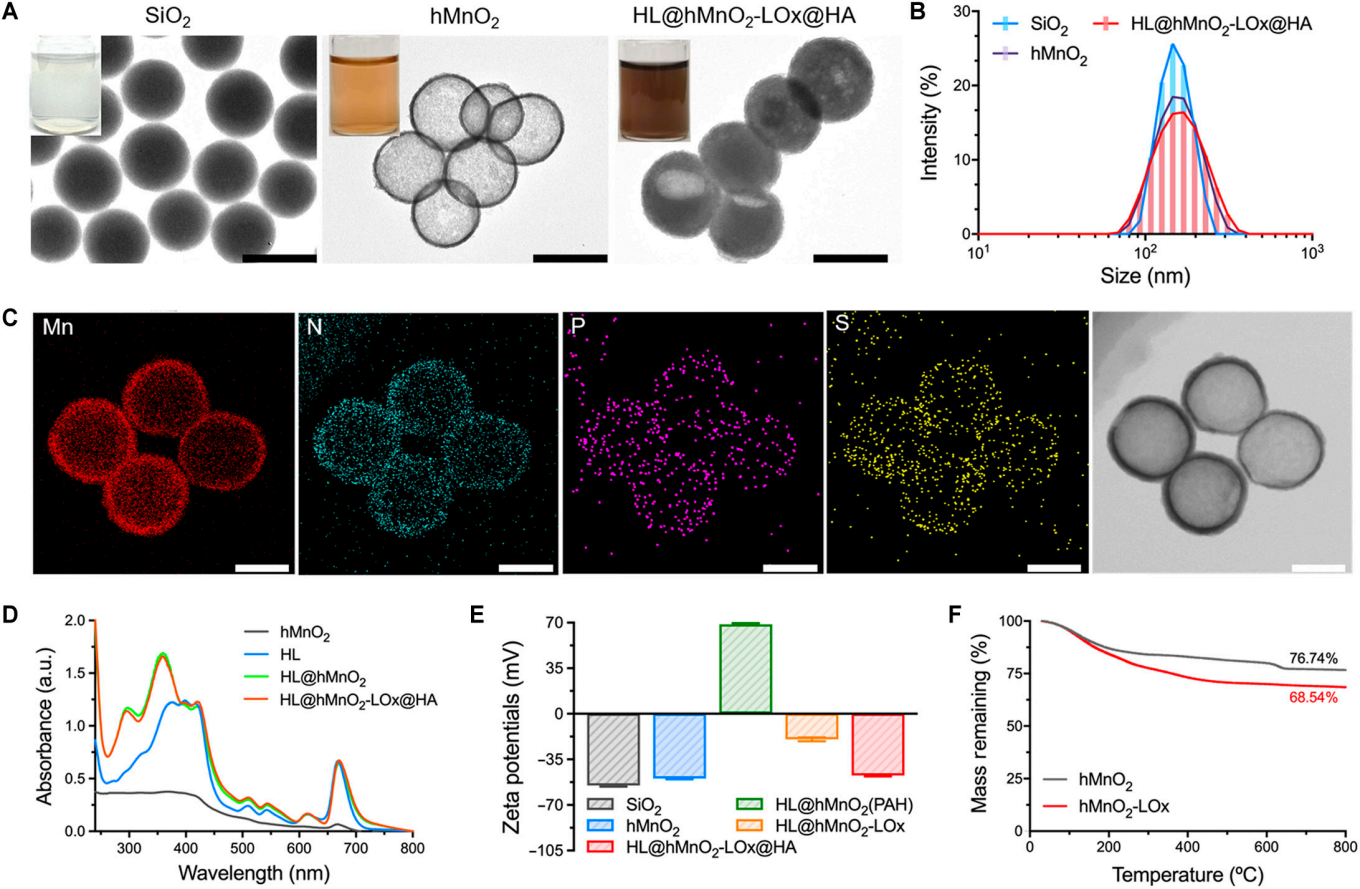
Characterizations of HL@hMnO_2_-LOx@HA. (A) TEM images of SiO_2_, hMnO_2_, and HL@hMnO_2_-LOx@HA. Scale bars: 200 nm. Insets are corresponding photographs of aqueous solutions. (B) Size distribution of SiO_2_, hMnO_2_, and HL@hMnO_2_-LOx@HA. (C) The element mapping images of hMnO_2_-LOx. Scale bars: 100 nm. (D) UV-vis-NIR spectra of hMnO_2_, HL, HL@hMnO_2_, and HL@hMnO_2_-LOx@HA. (E) Zeta potentials of SiO_2_, hMnO_2_, HL@hMnO_2_(PAH), HL@hMnO_2_-LOx, and HL@hMnO_2_-LOx@HA. (F) TG curves of hMnO_2_ and hMnO_2_-LOx.

To fabricate the HLMLH nanosystem, the hMnO_2_ nanoparticle was first synthesized according to the previously reported method [41]. Transmission electron microscope (TEM) images showed that the monodispersed silica nanoparticles are spheric and smooth (Fig. 2A), while the coating of MnO_2_ was relatively coarse (Fig. S1). After being etched with Na_2_CO_3_, the as-prepared hMnO_2_ nanoparticles had a uniform hollow and spheric morphology. The modification of LOx and HA had negligible influence on its morphology, but the cavity of hMnO_2_ turned nontransparent due to the encapsulation of HPPH and luminol. Dynamic light scattering measurement showed that the average diameter of the hMnO_2_ nanoparticle was about 165 nm, and the polydispersity index was 0.2841 (Fig. 2B). The decoration of LOx and HA increased its diameter to about 172 nm. The element mapping data indicated the existence of Mn in the shell and the loading of LOx on its surface (Fig. 2C). Ultraviolet (UV)-visible (vis)-near-infrared (NIR) spectra showed that HL@hMnO_2_- LOx@HA had a peak absorption at about 360 nm, indicating the effective encapsulation of HPPH (Fig. 2D). The LOx was loaded in the HLMLH nanosystem by electrostatic adsorption between an electronegative enzyme and electropositive poly(allylamine hydrochloride) (PAH) [42]. After loading LOx, the surface charge of the obtained HL@hMnO_2_-LOx returned negative (−23 mV) (Fig. 2E). After coating HA, the surface zeta potential further decreased to −47.3 mV. The optimized mass ratio of HPPH and hMnO_2_ was 7:2 (Fig. S2A). As calculated by the thermogravimetric (TG) analysis of hMnO_2_-LOx, the loading efficiency of LOx was about 8.2 wt% (Fig. 2F). The as-prepared HL@hMnO_2_-LOx@HA could be stable at 4°C within 7 d, indicating good stability (Fig. S2B).

### Chain reactions for the self-illuminated/-oxygenated PDT system

**Fig. 3.**
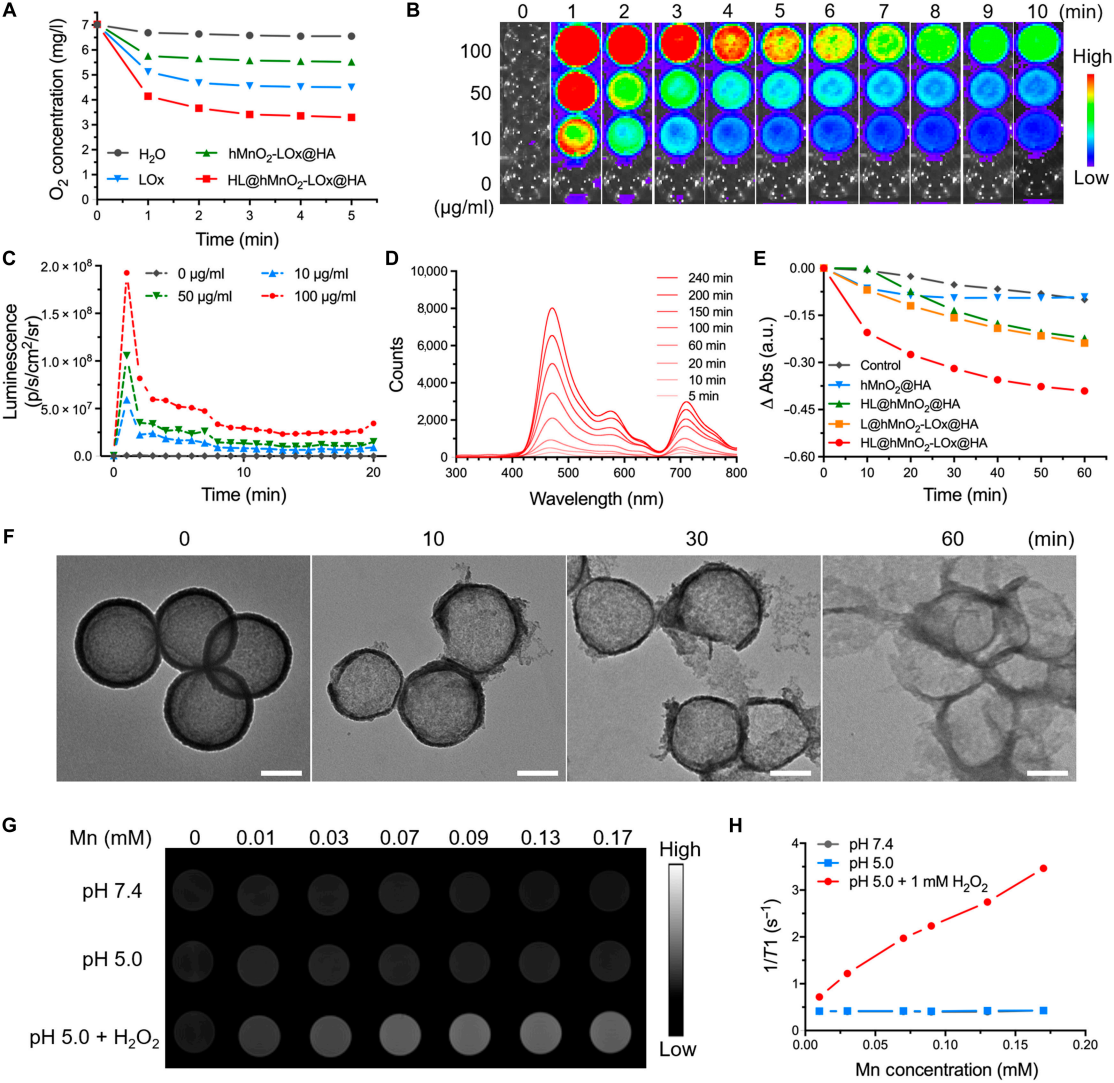
Chain reactions between HL@hMnO_2_-LOx@HA and LA. (A) The concentrations of O_2_ during the reaction between LA and indicated solutions (hMnO_2_: 10 μg/ml, LOx: 35 μg/ml). (B) Luminescence of HL@hMnO_2_-LOx@HA at various concentrations in the presence of 100 μM H_2_O_2_. (C) Corresponding luminescence signal intensities during the 10-min reaction between HL@hMnO_2_-LOx@HA and H_2_O_2_. (D) Time-resolved luminescent spectra of HL@hMnO_2_-LOx@HA in the presence of 10 mM LA. (E) ^1^O_2_ generated from the reaction between LA and indicated solutions (hMnO_2_: 10 μg/ml, Luminol: 10 μg/ml, LOx: 35 μg/ml), indicated by the absorption at 410 nm of a ^1^O_2_ probe, DPBF. (F) TEM images of HL@hMnO_2_-LOx@HA incubated in 100 μM H_2_O_2_ for 0, 10, 30, and 60 min. Scale bars: 100 nm. (G) *T*_1_-weighted MRI images of HL@hMnO_2_-LOx@HA at different concentrations in indicated PBS solutions. (H) Corresponding 1/*T*_1_ versus Mn concentration curves.

The chain reactions between LA and HL@hMnO_2_-LOx@HA were then investigated in vitro. After adding LA to both the H@hMnO_2_-LOx@HA (without luminol) and HL@hMnO_2_-LOx@HA aqueous solutions, H_2_O_2_ was continuously generated within 70 min owing to the catalytic reaction mediated by LOx (Fig. S3). However, the H_2_O_2_ amount was relatively lower in the HL@hMnO_2_-LOx@HA group, indicating that luminol in the nanosystem could also react with the generated H_2_O_2_. Theoretically, the generated H_2_O_2_ will then react with hMnO_2_ to produce O_2_ and improve the hypoxic state of TNBC. To verify this assumption, the O_2_ content of LA aqueous solutions was measured using a dissolved oxygen meter. Results showed that in the absence of LA and luminol, 100 μM H_2_O_2_ reacted with hMnO_2_ and largely increased the O_2_ concentration within 1 min from 3.32 mg/ml to higher than 10 mg/ml. In the presence of luminol, the O_2_ concentration rose in the first 30 s and then dropped slowly and maintained higher than 7 mg/ml within 6 min, indicating that the generated O_2_ is enough for chemiluminescence-excited PDT. Comparatively, the LOx-LA reaction only decreased the O_2_ concentration within 1 min from 7.01 to 4.15 mg/ml (Fig. 3A), significantly lower than that generated from the reaction between endogenous H_2_O_2_ and hMnO_2_ (the HL@hMnO_2_-LOx@HA + H_2_O_2_ group in Fig. S4).

In the presence of H_2_O_2_ at a physiological concentration (100 μM), luminol immediately emitted strong blue luminescence in 1 min and lasted for more than 5 min, guaranteeing sufficient excitation of surrounding HPPH (Fig. 3B to C). During the reaction, the typical FL emission of HPPH at about 707 nm rapidly increased as the characteristic luminescent emission of luminol at about 460 nm gradually decreased as the reactants were exhausted (Fig. S5). Similarly, in the presence of 11 mM LA (close to physiological concentration), luminol emitted moderate blue luminescence, slighter than that emitted from the mixture of nanoparticles at a similar concentration (50 μg/ml) and 100 μM H_2_O_2_. The luminescence intensity peaked in 2 min and lasted for about 10 min (Fig. S6). Characteristic luminescent emissions of luminol and HPPH were also observed in the mixed solution of HL@hMnO_2_-LOx@HA and LA at a physiological concentration (10 mM) (Fig. 3D). Together, these results indicated that both intratumoral LA and H_2_O_2_ could trigger the self-acting PDT process.

Then, 1,3-diphenylisobenzofuran (DPBF) was used to assess the production of ^1^O_2_. As shown in Fig. 3E, while HL@hMnO_2_@HA could also produce a moderate amount of ^1^O_2_ when incubated with LA for 60 min, the HL@hMnO_2_-LOx@HA group produced the most amount of ^1^O_2_, indicated by the sharp absorption decrease of DPBF (Fig. S7). As mentioned before, the H_2_O_2_ generated from LA-LOx catalytic reaction could degrade the hMnO_2_ nanoparticle. TEM images in Fig. 3F consolidated the gradual degradation of the hMnO_2_ nanoparticle within 60 min of incubation with H_2_O_2_. This reaction could produce Mn^2+^ ions, an effective *T*_1_ contrast agent for MRI. The lighter *T*_1_-weighted MR images of HL@hMnO_2_-LOx@HA ([Mn] = 0, 0.01, 0.03, 0.07, 0.09, and 0.17 mM) incubated with H_2_O_2_ at pH 5.0 were then captured (Fig. 3G). The slope, as given by the *r*_1_ value, was evaluated to be 16.53 mM^−1^s^−1^, higher than that of pH 7.4 group (0.076 mM^−1^s^−1^) and pH 5.0 group (0.069 mM^−1^s^−1^) (Fig. 3H). The degradation rate of the hMnO_2_ shell was slower in 10 mM LA than in 1 mM H_2_O_2_. The complete degradation of nanoparticles took about 4 h (Fig. S8A). Without LA and H_2_O_2_, hMnO_2_ hardly decomposed into Mn ions (Fig. S8B), and the *r*_1_ value of the control group was only 0.080 mM^−1^s^−1^ (Fig. S8C), lower than that of the LA group (5.856 mM^−1^s^−1^). These results indicate that an H_2_O_2_-activated MRI could be performed to instruct the self-acting PDT process by revealing the generation of H_2_O_2_.

### Self-acting PDT in vitro

**Fig. 4.**
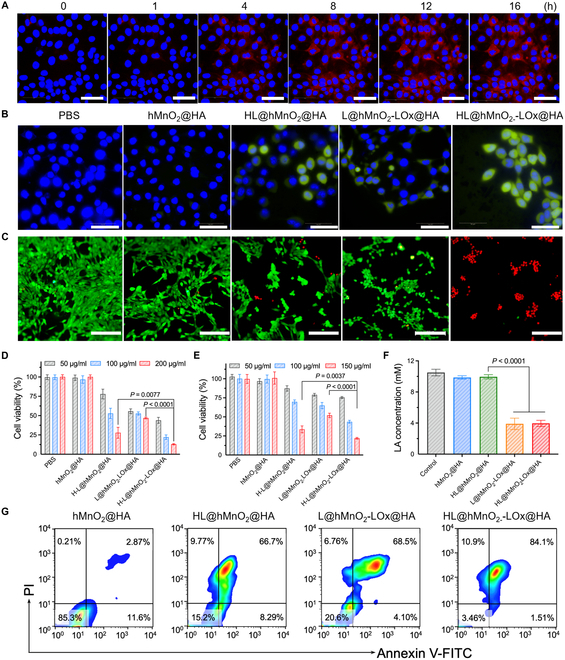
In vitro self-acting PDT. (A) FL images of 4T1 cells incubated with HL@hMnO_2_-LOx@HA for 0, 1, 4, 8, 12, and 16 h, respectively (hMnO_2_: 200 μg/ml). Scale bars: 50 μm. (B) ROS generation from 4T1 cells after different treatments, as indicated by the FL of a ROS probe, H_2_DCFDA. Scale bar: 50 μm. (C) Live/dead analysis of 4T1 tumor cells after various treatments, indicated by the FL of calcium AM (CA, green) and PI (red), respectively. Scale bar: 500 μm. (D) Relative viability of normoxic 4T1 cells treated with PBS, hMnO_2_@HA, HL@hMnO_2_ @HA, L@hMnO_2_-LOx@HA, and HL@hMnO_2_-LOx@HA at different concentrations, respectively. (E) Relative viability of hypoxic 4T1 cells treated with PBS, hMnO_2_@HA, HL@hMnO_2_ @HA, L@hMnO_2_-LOx@HA, and HL@hMnO_2_-LOx@HA at different concentrations, respectively. (F) Intracellular LA concentrations of 4T1 cells after indicated treatments. (G) Representative flow cytometric profiles of cell apoptosis (using an Annexin V-fluorescein isothiocyanate/PI apoptotic kit) within normoxic 4T1 tumors after 12 h of incubation with hMnO_2_@HA, HL@hMnO_2_@HA, L@hMnO_2_-LOx@HA, and HL@hMnO_2_-LOx@HA, respectively. *n* = 5. Data are means ± SD. Statistical analysis was conducted using a 2-tailed Student *t* test.

The above results demonstrated the efficient chain reactions among HL@hMnO_2_-LOx@HA and LA that produced O_2_ and exciting HPPH without extra fiber. Subsequently, this self-illuminated/-oxygenated PDT process mediated by HL@hMnO_2_-LOx@HA was investigated in vitro. The significantly increased fluorescence (FL) intensity of HPPH in 4T1 tumor cells under an FL microscope confirmed the intracellular incorporation of HL@hMnO_2_-LOx@HA (Fig. 4A). The semiquantitative analysis showed that most of the nanoparticles were incorporated in cells within 10 h of incubation (Fig. S9). Bio-TEM images further demonstrated the efficient cellular uptake of HL@hMnO_2_-LOx@HA (Fig. S10). Uptake blocking experiment using free HA demonstrated the active targeting capability of HA-decorated HL@hMnO_2_-LOx@HA nanoparticles toward 4T1 cells (Fig. S11). To predict the efficacy of self-acting PDT in vitro, a commercial probe of reactive oxygen species (ROS), H_2_DCFDA, was used to detect the intracellular generation of ROS including ^1^O_2_, the main ROS for PDT. Once encountering ROS in the cytoplasm, the probe would emit strong green FL. Without HPPH, cells treated with L@hMnO_2_-LOx@HA also emitted slight green FL because H_2_O_2_ generated from LA-LOx catalytic reaction could also be detected by the ROS probe (Fig. 4B). Without LOx, cells treated with HL@hMnO_2_@HA also emitted moderate green FL because intracellular H_2_O_2_ could also excite the luminol and produce ^1^O_2_ via CRET. After being treated with HL@hMnO_2_-LOx@HA, every 4T1 cell exhibited intense green FL, indicating that a large amount of ^1^O_2_ was produced (Fig. S12A). Strong green FL was also observed in HL@hMnO_2_-LOx@HA-treated 4T1 cells under hypoxic conditions (Fig. S12B and C).

The PDT efficacy of HL@hMnO_2_-LOx@HA was then semiquantified by staining the live/dead cells using calcium AM/propidium iodide (PI) (Fig. 4C). Similar to the results of ROS staining, cells treated with both HL@hMnO_2_@HA and L@hMnO_2_-LOx@HA only exhibited slight red FL (dead cells), while almost no live cell remained after treatment with HL@hMnO_2_-LOx@HA. Then, [3-(4,5-dimethylthiazol-2-yl)-2,5-diphenyltetrazolium bromide] (MTT) assay was conducted to quantitatively evaluate the efficacy of self-acting PDT. Both phosphate buffer solution (PBS) and hMnO_2_@HA caused negligible cell death, indicating good biosafety of the nanocarrier. The treatment of HL@hMnO_2_-LOx@HA killed 83.4% of 4T1 cells, while that of HL@hMnO_2_@HA and L@hMnO_2_-LOx@HA killed 77.7% and 67.8%, respectively (Fig. 4D). To confirm the self-oxygenation mediated by HL@hMnO_2_-LOx@HA, cells were treated with different groups in a hypoxic incubator. Although the pO_2_ decreased to approximately 5%, self-oxygenated PDT maintained high PDT efficacy and killed more than 87% of 4T1 cells (Fig. 4E). These results consolidated the oxygen-supplementing capability of HL@hMnO_2_-LOx@HA during the PDT process. In addition to PDT, the depletion of LA also contributed to the excellent cell-killing effect. Both L@hMnO_2_-LOx@HA and HL@hMnO_2_-LOx@HA groups showed a obviously decreased LA content from about 10 to 4 mM in 4T1 cells (Fig. 4F). Annexin V-PI costaining assay showed that self-acting PDT mainly caused late-stage cell apoptosis (84.1%) (Fig. 4G).

### Multimodal imaging in vivo

**Fig. 5.**
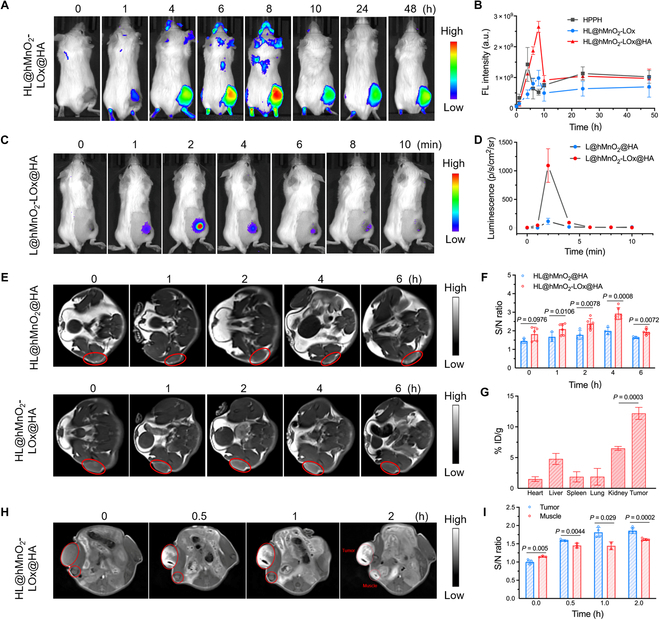
In vivo FL/BL/MR imaging. (A and B) In vivo FL images (A) and corresponding semiquantitative analysis (B) of subcutaneous 4T1 tumor-bearing mice 0, 1, 4, 6, 8, 10, 24, and 48 h post intravenous injection of HL@hMnO_2_-LOx@HA. HPPH: 10 mg/kg. (C and D) In vivo chemiluminescent imaging (C) and corresponding semiquantitative analysis (D) of subcutaneous 4T1 tumor-bearing mice 0, 1, 2, 4, 6, 8, and 10 min post intratumoral injection of HL@hMnO_2_-LOx@HA. HPPH: 10 mg/kg. (E and F) *T*_1_-weighted MRI (E) and corresponding S/N ratios (F) of the 4T1 tumor (indicated by red circles) 0, 1, 2, 4, and 6 h post intravenous injection of HL@hMnO_2_-LOx@HA. HPPH: 10 mg/kg. (G) Mn distribution in heart, liver, spleen, lung, kidney, and tumor of 4T1 tumor-bearing mice 12 h post intravenous injection of HL@hMnO_2_-LOx@HA. (H and I) *T*_1_-weighted MRI (H) and corresponding S/N ratios (I) of the 4T1 tumor and muscle (indicated by red circles) 0, 0.5, 1, and 2 h post intratumoral or intramuscular injection of HL@hMnO_2_-LOx@HA. HPPH: 10 mg/kg. *n* = 3. Data are means ± SD. Statistical analysis was conducted using a 2-tailed Student *t* test.

Next, the FL/MR dual-modal imaging performance of HL@hMnO_2_-LOx@HA was evaluated on the subcutaneous 4T1 tumor-bearing mice. The maximum diameter of HL@hMnO_2_-LOx@HA was less than 200 nm (Fig. 2A and B), a suitable size for the enhanced permeation and retention effect [43]. While free HPPH was distributed all over the body, HL@hMn_2_-LOx passively targeted the 4T1 tumor via the enhanced permeation and retention effect and its FL signal peaked at 8 h after intravenous injection (Fig. S13). HA could selectively bind with CD44 highly expressed on the cell membrane of TNBC cells [44]. HA coating endowed the HL@hMnO_2_-LOx@HA nanoparticle with active targeting capability, which achieved markedly higher tumor accumulation than L@hMn_2_-LOx (Fig. 5A and B). Once intratumorally injected with HL@hMnO_2_-LOx@HA, luminol emitted more intense luminescence than that with L@hMnO_2_-LOx, further indicating the higher tumor accumulation (Fig. 5C and Fig. S14). Although the luminescence signal turned weak quickly due to the interference from tissues (e.g., blood, skin, fur), the ex vivo luminescence signals in the tumors remained visible 60 min after injection, indicating sufficient excitation of HPPH for in vivo PDT (Fig. S15). As mentioned before, the hMnO_2_ nanoparticle would be degraded by H_2_O_2_ in an acidic environment and generated Mn^2+^ for *T*_1_-weighted MRI (Fig. 3G and H). After intravenous injection, the tumor *T*_1_-MR signals from both HL@hMnO_2_-LOx@HA and HL@hMnO_2_@HA increased and peaked at ~4 h (Fig. 5E). The existence of LOx enhanced the MR signal due to the generation of extra H_2_O_2_ from LOx-catalyzed LA oxidation (Fig. 5F). The quantitative analysis of the tissue distribution of HL@hMnO_2_-LOx@HA was performed using inductively coupled plasma–mass spectrometry (ICP-MS). The content of Mn^2+^ in the tumor was observably higher than that in the other major organs, further indicating the high tumor accumulation of HL@hMnO_2_-LOx@HA (Fig. 5G). Comparatively, the muscle *T*_1_-MR signals of HL@hMnO_2_-LOx@HA were markedly lower than that in the tumor, because intratumoral LA and H_2_O_2_ as well as the acidic tumor microenvironment contributed to the generation of Mn^2+^ (Fig. 5H and I). The 3 factors also contributed to the chemiluminescence of luminol that excited HPPH for PDT, so activatable *T*_1_-MRI could be used to monitor the efficacy of self-acting PDT.

### Self-acting PDT in vivo

**Fig. 6.**
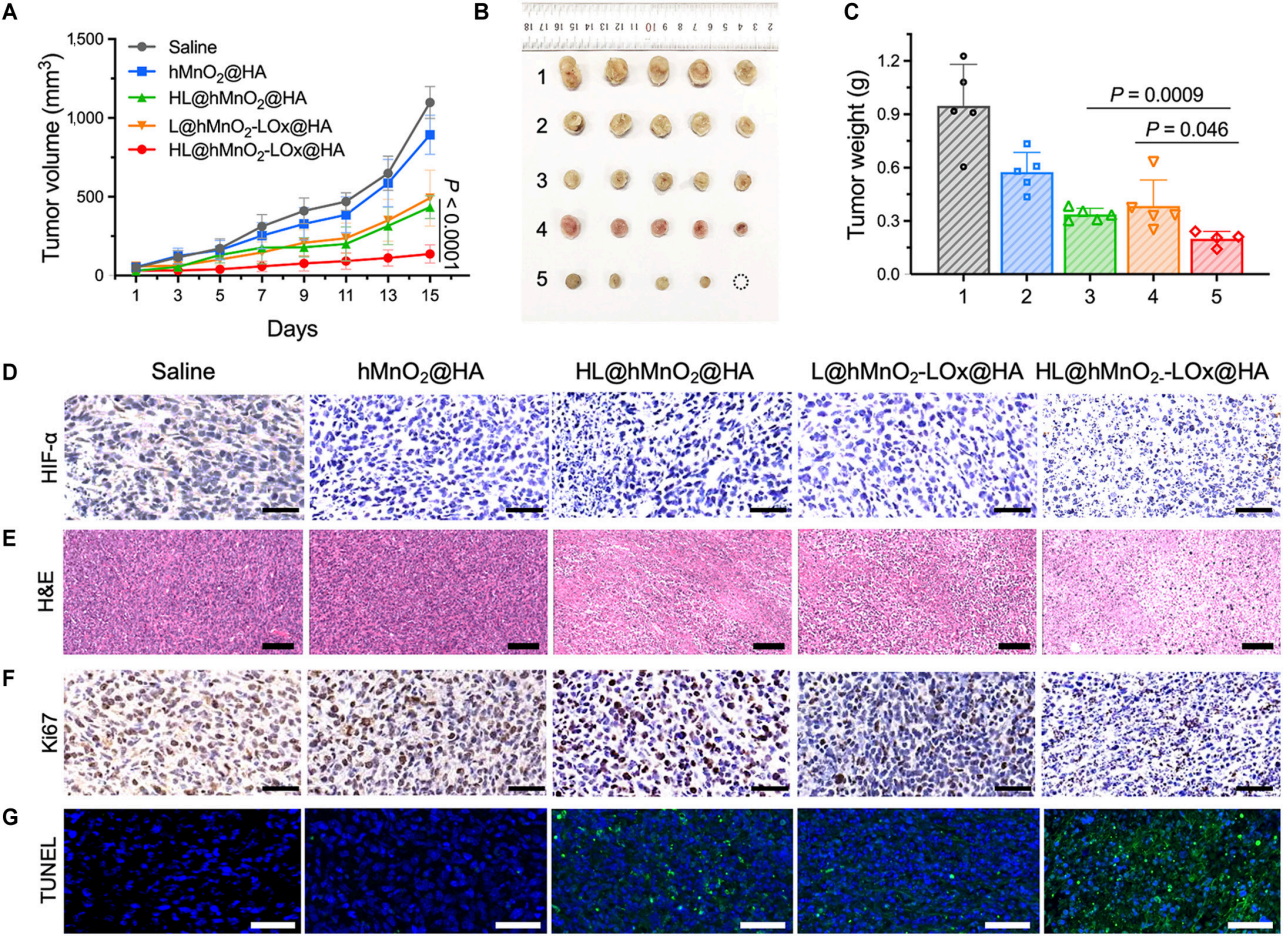
In vivo self-acting PDT. (A) Tumor growth curves of subcutaneous 4T1 tumor-bearing mice after receiving indicated treatments. *n* = 5. (B and C) Digital photos (B) and quantified tumor weight (C) of excised tumors at day 15 after indicated treatments: 1) saline, 2) hMnO_2_@HA, 3) HL@hMnO_2_@HA, 4) L@hMnO_2_-LOx@HA, and 5) HL@hMnO_2_-LOx@HA. (D) Representative IHC images of HIF-1α expression in tumor sections collected 15 d after indicated treatments. Scale bars: 100 μm. (E) Representative H&E staining for pathological changes in tumor sections collected 15 d after indicated treatments. Scale bars: 200 μm. (F) Representative IHC images of Ki67 expression in tumor sections collected 15 d after indicated treatments. Scale bars: 100 μm. (G) Representative FL images of TUNEL staining of tumor sections collected 15 d after indicated treatments. Scale bars: 100 μm. Data are means ± SD. Statistical analysis was conducted using one-way analysis of variance (ANOVA) with Tukey’s test.

The prerequisite of effective PDT on TNBC tumors is to conquer the limits of light penetration and tumor hypoxia. With the guidance of dual-modal MRI/FLI, HL@hMnO_2_-LOx@HA-mediated self-illuminated/-oxygenated PDT represents a promising strategy to treat TNBC. To investigate the in vivo efficacy of self-acting PDT, HL@hMnO_2_-LOx@HA was intravenously injected into a 4T1 TNBC xenograft model with a tumor volume of ≈ 70 mm^3^. Since HPPH was excited by blue chemiluminescence emitted from luminol, there was no laser irradiation of the traditional PDT process. The tumor growth was then monitored for 15 d, and the tumor growth inhibition (TGI) rates of each treatment group were calculated following a reported method [45]. Tumors in the mice treated with the pure carrier hMnO_2_@HA grew slightly lower than that of the saline group, and the average TGI rate was 23.1% (Fig. 6A). The tumor inhibition effect of the carrier was probably because that hMnO_2_ could react with H^+^ and H_2_O_2_ to improve the acidic and oxidative tumor microenvironment. The HL@hMnO_2_@HA group (without LOx) and the L@hMnO_2_-LOx@HA group (without HPPH) showed similar tumor suppression efficacy, and their TGI rates were 62.4% and 61.3%, respectively. The antitumor effect of HL@hMnO_2_@HA was mainly attributed to the intratumoral H_2_O_2_-triggered chemiluminescence excited PDT, and that of L@hMnO_2_-LOx@HA was mainly because of the LOx-mediated LA depletion and production of biotoxic H_2_O_2_. Among all these groups, HL@hMnO_2_-LOx@HA achieved the most significant tumor inhibition efficacy with a TGI rate of 80.2% owing to the LA/LOx-triggered self-illuminated/-oxygenated PDT. Compared to HL@hMnO_2_@HA, HL@hMnO_2_-LOx@HA provided more H_2_O_2_ required for chemiluminescence via LOx-catalyzed LA oxidization. Compared to L@hMnO_2_-LOx@HA, HL@hMnO_2_-LOx@HA introduced HPPH to achieve chemiluminescence-excited PDT. Digital photos showed that one tumor was eliminated (Fig. 6B) and most of the tumors in this group remained at the original size (Fig. S16), only about one-eighth the weight of the saline group at day 15 (Fig. 6C). Hypoxia induces the expression of hypoxia-inducible factor 1α (HIF-1α) [46]. Immunohistochemical (IHC) analysis indicated that HIF-1α expression was markedly down-regulated in the tumors of mice treated with hMnO_2_-contained nanoparticles (Fig. 6D and Fig. S17). Eight hours after the treatment, intratumoral LA concentration decreased from 19.7 to 15.4 μmol/g, demonstrating the significant LA consumption (Fig. S18).

To assess the morphological changes of tumor tissues, hematoxylin and eosin (H&E) staining was performed on tumor slices collected 15 d after different treatments. Among the 4 treatment groups, the tumor tissue of the HL@hMnO_2_-LOx@HA group exhibited the most severe plasmatorrhexis, nucleus shrinkage, karyorrhexis, and the largest intercellular spaces (Fig. 6E). IHC analysis also showed that the expression of Ki67, a marker of tumor proliferation, was markedly down-regulated 15 d after the treatment of HL@hMnO_2_-LOx@HA (Fig. 6F). Terminal deoxytransferase digoxigenin-dUTP nick end labeling (TUNEL) staining showed that tumor cells in the HL@hMnO_2_-LOx@HA group exhibited the highest level of cell apoptosis, the main cell death type caused by PDT and excessive H_2_O_2_ (Fig. 6G). No obvious decrease in body weight was observed within 15 d after various treatments, indicating that HL@hMnO_2_-LOx@HA did not cause acute systemic toxicity (Fig. S19). The serum chemistry results further confirmed that HL@hMnO_2_-LOx@HA had no acute toxicity to the liver or kidney (Fig. S20). The tissue morphology of major organs in all these treatment groups remained normal (Fig. S21). In addition, no obvious hemolysis was in the blood containing 400 μg/ml HL@hMnO_2_-LOx@HA (Fig. S22). These results indicate good biosafety of this self-acting PDT nanosystem.

## Discussion

In summary, an LA-fueled self-acting PDT nanosystem was fabricated to perform self-illuminated/-oxygenated PDT against TNBC. The as-prepared hMnO_2_-LOx@HA nanoparticle could selectively accumulate in the tumor and deplete intratumoral LA via a LOx-catalyzed oxidation reaction. Meanwhile, the generated H_2_O_2_ reacted with luminol and emitted blue luminescence, which could last for 60 min to guarantee the sufficient excitation of HPPH for PDT. H_2_O_2_ degraded the hMnO_2_ nanoparticle in the acidic microenvironment and produced O_2_ to supplement the source of ^1^O_2_ for PDT. Moreover, the hMnO_2_ was degraded to Mn^2+^ and generated strong *T*_1_-MR signals 4 h after intravenous injection in the 4T1 tumors, thereby monitoring the PDT process. Mn^2+^ catalyzed the decomposition of H_2_O_2_ to produce •OH and enhanced the chemiluminescence. More than 87% of 4T1 cells were killed by self-acting PDT in vitro, and the TGI rate was 80.2% in a 4T1 TNBC xenograft model. Therefore, this nanosystem is promising to simultaneously conquer the limits of light penetration and tumor hypoxia for traceable PDT against TNBC tumors as well as LA overproduction.

## Materials and Methods

### Materials

2-(l-hexoxy)-2-ethyl derivative of pyromellitic chloride-&-(HPPH) was supplied by MedChemExpress (Shanghai, China). Ethyl orthosilicate, diphenyl benzofuran (DPBF), penicillin-streptomycin, trypsin, phosphate buffer (PBS), fetal bovine serum, and 4′,6-diamidino-2-phenylindole were from ThermoFisher Scientific (Shanghai, China). LOx, Dulbecco’s modified Eagle’s medium (DMEM), thiazole blue (MTT), and 2′,7′-dichlorodihydrofluorescein diacetate (DCFH-DA) were from Sigma-Aldrich (Shanghai, China). Polyallylamine salt (PAH) and HA were provided by Macklin (Shanghai, China). Calcein-AM and PI was from Biolegend (USA).

### Preparation of HL@hMnO_2_-LOx@HA

The mass ratios of HPPH, Luminol, and hMnO_2_ were 7:5:2. Seventy milligrams of HPPH and 50 mg of Luminol in 0.5 ml of dimethyl sulfoxide buffer was dispersed in 2 ml of hMnO_2_ nanoparticle aqueous solution (1 mg/ml) and stirred for 24 h at 4 °C. The HL@hMnO_2_ nanoparticles were then collected by centrifugation (12,000 rpm, 25 min) and dispersed in ultrapure water. Afterward, the HL@hMnO_2_ nanoparticles were added to 2 ml of PAH solution (10 mg/ml), stirred for 30 min, and washed with ultrapure water to obtain positively charged nanoparticles. A certain amount of LOx (500 μg for animal experiments and 35 μg for cell experiments) was mixed with the PAH-modified nanoparticles and shaken for 15 min. HA solution (10 mg/ml of 1 ml) was added to the above solution, and shaking was continued for 15 min to stabilize the nanoparticles (HL@hMnO_2_-LOx@HA) before being washed with ultrapure water.

### Characterization of HL@hMnO_2_-LOx@HA

The morphology of the HL@hMnO_2_-LOx@HA was observed by transmission electron microscopy (HT7700 TEM; Hitachi, Japan). The constituent elements were analyzed by an inductively coupled plasma mass spectrometer (NexION 300X; PerkinElmer, USA). A Malvern Zetasizer Nano ZS Particle and Zeta Potential Analyzer (DTS 1060, Malvern, UK) examined its hydrated particle size and zeta potential. Its absorption spectrum was detected by an Agilent Cary 60 UV-vis-NIR spectrophotometer (Agilent Technologies, Santa Clara, USA). The encapsulation efficiency and drug loading of HPPH and LOx were measured according to the formula as follows: Encapsulation efficiency (%) = Mass of drugs in the nanoparticles (g)/Mass of nanoparticles × 100%. Drug loading (%) = Mass of drugs in the nanoparticles (g)/Mass of drug added in the nanoparticles × 100%. For in vitro imaging, the concentration of Mn was measured using the inductively coupled plasma mass spectrometer.

### Acid-responsive degradation of HL@hMnO_2_-LOx@HA

HL@hMnO_2_-LOx@HA aqueous solution (2 ml) (0.5 mg/ml) was placed in a dialysis bag (molecular weight cutoff = 3,500 Da), and dialyzed against PBS buffer (pH 7.4) or citric acid buffer (pH 5.0) at 37 °C. Dialysis buffer (2 ml) was removed at regular intervals to detect Mn^2+^ by inductively coupled plasma. After dialysis, the morphology of the remained HL@hMnO_2_-LOx@HA nanoparticles was observed by a TEM.

### Cellular uptake

4T1 cells were inoculated in 96-well plates at a density of 5 × 10^3^ per well and incubated overnight. Different concentrations of HPPH, HL@hMnO_2_-LOx@HA, and HL@hMnO_2_-LOx@HA plus HA were added to the DMEM, respectively. The cells were then incubated for 24 h, followed by measurement of intracellular fluorescence intensity after uptake in each group using a high-throughput imaging system (Operetta CLS; PerkinElmer, Hamburg, Germany).

### Intracellular ROS detection

Intracellular ROS was detected using the ROS green fluorescent dye DCFH-DA (Ex/Em: 495/529 nm). 4T1 cells were inoculated at a density of 7 × 10^4^ per well in 12-well plates and incubated overnight. The medium was then changed to a serum-free medium containing hMnO_2_, HL@hMnO_2_, L@hMnO_2_-LOx@HA, and HL@hMnO_2_-LOx@HA. Then, the DCFH-DA was added and incubated for 30 min. The cells were washed 3 times with PBS, and a serum-free medium was added to monitor green fluorescence within the cells under an inverted fluorescent microscope (Eclipse Ti2 Series-Nikon, Japan).

### Cytotoxicity

The 4T1 cells were inoculated in 96-well plates at a density of 5 × 10^3^ per well and incubated overnight. Afterward, HPPH, hMnO_2_, HL@hMnO_2_, L@hMnO_2_-LOx@HA, and HL@hMnO_2_-LOx@HA at various concentrations were added and incubated for 12 h, respectively. To measure the cell viability, the DMEM was replaced with 100 μl of fresh medium containing MTT (1 mg/ml). After incubation for 4 h, the medium containing MTT was replaced with 150 μl of dimethyl sulfoxide. The absorbance at 490 nm of the cell plate was measured using an enzyme marker. Cell viability (%) = (OD_490 nm_ of sample/OD_490 nm_ of control) * 100%. To visualize the dead/live tumor cells, calcein-AM (4 μM, green) and PI (4 μM, red) were added and incubated for 30 min, respectively. A Nikon Eclipse Ti inverted fluorescence microscope (Nikon Canada, Mississauga, Canada) was used to observe the fluorescence of cells. To evaluate the cell apoptosis and necrosis, the cells were digested by trypsin and collected for the staining of Annexin V-fluorescein isothiocyanate and PI before analysis using flow cytometry (FACSAria III; BD Biosciences, San Jose, CA, USA).

### In vivo FLI

When the tumor volume reached 200 mm^3^, HPPH, HL@hMnO_2_-LOx, and HL@hMnO_2_-LOx@HA (HPPH: 10 mg/kg) were intravenously injected in the 4T1 tumor-bearing mice. Fluorescence images of mice were collected by using an IVIS Spectrum system (Caliper Life Sciences, Hopkinton, MA) 0, 1, 2, 4, 6, 8, 10, 12, 24, 48, and 72 h after injection, respectively.

### In vivo MRI

After tail vein injection of HL@hMnO_2_@HA and HL@hMnO_2_-LOx@HA (HPPH: 10 mg/kg) in mice, MRI images of mice were collected by a 3.0-T magnetic resonance system (MAGNETOM Skyra, Siemens Healthcare, Forccheim, Germany) 0, 1, 2, 4, and 6 h after injection.

### In vivo PDT

Once the tumor volume reached approximately 70 mm^3^, the tumor-bearing mice were randomized into 5 groups: (a) saline, (b) MnO_2_, (c) HL@hMnO_2_@HA, (d) L@hMnO_2_-LOx@HA, and (e) HL@hMnO_2_-LOx@HA. For photodynamic treatment, saline, MnO_2_, HL@hMnO_2_@HA, L@hMnO_2_-LOx@HA, and HL@hMnO_2_-LOx@HA (HPPH: 10 mg/kg) were intravenously injected into the tumor-bearing mice twice every week, respectively. The tumor length and width of the mice were measured using a vernier caliper to calculate the tumor volume until the tumor volume exceeded 2,000 mm^3^. The tumor volume was calculated according to the following formula: volume = width^2^ × (length/2). If the tumor volume of mice exceeds 2,000 mm^3^, they will be euthanized by an overdose of chloral hydrate, and their survival time will be recorded. Tumor growth inhibition rate (%TGI) was calculated according to the following equation: %TGI = {1 − (*V*_t15_/*V*_t0_)/ (*V*_c15_/*V*_c0_)} × 100. *V*_c15_ and *V*_t15_ are the mean volumes of the saline and treated groups at day 15, respectively. *V*_c0_ and *V*_t0_ are the mean volumes of saline and treated groups at day 0, respectively.

### Histological analysis

Fourteen days after various treatments, all mice were executed and the major organs (heart, liver, spleen, lung, and kidney) and tumors of representative mice were collected and fixed in 4% paraformaldehyde solution; then, tissue paraffin sections were made and stained for H&E. Ki67, HIF-1α, and TUNEL staining was also performed on tumor sections. Histological changes were observed using TEKSQRAY Slide Scan System SQS1000 (Shengqiang Technology Co., Ltd., Shenzhen, China).

### Statistical analysis

The data were shown as mean ± SD. Statistical differences were established using an unpaired 2-tailed Student *t* test or one-way analysis of variance (ANOVA) followed by Fisher’s LSD test in GraphPad Prism 8.2. *P < 0.05, **P < 0.01, ***P < 0.001.

## Supplementary Materials

Supplementary 1 Experimental Procedures Figs. S1 to S22
