# Supplementary material for: Traceable Lactate-Fueled Self-Acting Photodynamic Therapy against Triple-Negative Breast Cancer
Source: Research (Wash D C). 2024 Jan 17;7:0277. doi: 10.34133/research.0277 (PMC12326368; doi:10.34133/research.0277)
Supplement: Supplementary 1 — Experimental Procedures Figs. S1 to S22 [file research.0277.f1.docx]

Traceable Lactate-Fueled Self-Acting Photodynamic Therapy Against Triple-Negative Breast Cancer

*Yifan Zhang^#^, Guangle Feng^#^, Ting He, Min Yang, Jing Lin, Peng Huang**

Marshall Laboratory of Biomedical Engineering, International Cancer Center, Laboratory of Evolutionary Theranostics (LET), Guangdong Key Laboratory for Biomedical Measurements and Ultrasound Imaging, School of Biomedical Engineering, Shenzhen University Health Science Center
Shenzhen, 518060 (China)

# These authors contributed equally to this work.
E-mail: peng.huang@szu.edu.cn

Experimental Procedures

**Preparation of hMnO_2_.** First, monodisperse silica nanoparticles were synthesized by adding 4 mL of tetraethyl orthosilicate (TEOS) to the aqueous solution of 50 mL of ethanol, 10 mL of water, and 1 mL of ammonia at room temperature for 14 h. The silica nanoparticles were then collected by centrifugation at 12,000 rpm for 25 min and used as a template to resuspend 980 mg of manganese permanganate (KMnO_4_) in 30 mL of water. Manganese permanganate (KMnO_4_) was sonicated for 30 min and the KMnO_4_ was reduced to MnO_2_ by the unreacted organic silica present on these freshly prepared silica nanoparticles to produce SiO_2_ nanoparticles with a uniform MnO_2_ layer growing on the surface (MnO_2_@SiO_2_). Hollow mesoporous MnO_2_ (hMnO_2_) nanoparticles were obtained by incubating the above MnO_2_@SiO_2_ nanoparticles with 2 M of Na_2_CO_3_ for 1~2 h.

**In vitro detection of ^1^O_2_.** 1,3-diphenylisobenzofuran (DPBF) was used to assess the production of ^1^O_2_. The aqueous solutions of hMnO_2_ (10 μg/mL), HL@hMnO_2_ (10 μg/mL hMnO_2_, 10 μg/mL HL), L@hMnO_2_-LOx@HA (10 μg/mL hMnO2, 10 μg/mL Luminol, 35 μg/mL LOx) and HL@hMnO_2_-LOx@HA (10 μg/ mL hMnO_2_, 10 μg/mL HL, 35 μg/mL LOx) were added with 10 μL DPBF (2 mg/mL) probe and 50 mM LA. The absorption spectra of DPBF were continuously recorded using a UV-vis-NIR spectrophotometer (Agilent Technologies, Santa Clara, USA) for 60 min.

**In vitro oxygen consumption.** To monitor the oxygen consumption, hMnO_2_ (10 μg/mL), HL@hMnO_2_ (10 μg/mL hMnO_2_, 10 μg/mL HL), L@hMnO_2_-LOx@HA (10 μg/mL hMnO_2_, 10 μg/mL Luminol, 35 μg/mL LOx) and HL@hMnO_2_-LOx@HA (10 μg/ mL hMnO_2_, 10 μg/mL HL, 35 μg/mL LOx) were added to the lactic acid aqueous solution (3 mL, 50 mM), respectively. Then the oxygen content in the solution was measured using a portable dissolved oxygen meter (JPBJ-608; Rex, Shanghai, China).

**In vitro H_2_O_2_ generation.** In vitro oxygen consumption: To monitor the oxygen consumption, hMnO_2_ (10 μg/mL), HL@hMnO_2_ (10 μg/mL hMnO_2_, 10 μg/mL HL), L@hMnO_2_-LOx@HA (10 μg/mL hMnO_2_, 10 μg/mL Luminol, 35 μg/mL LOx) and HL@hMnO_2_-LOx@HA (10 μg/ mL hMnO_2_, 10 μg/mL HL, 35 μg/mL LOx) were added to the lactic acid aqueous solution (3 mL, 50 mM), respectively. 50 µL above samples or standard substances were added to a 96-well plate, followed by the addition of 100 μL of hydrogen peroxide detection reagent. The plate was gently shaken and the peak absorption at 540-570 nm was then immediately measured using a fluorescence plate reader (Synergy H1; BioTek, VT, USA).

**Tumor model.** Four-week-old female BALB/c mice were purchased from the Guangdong Experimental Animal Centre, and the animal experimental method was approved by the Experimental Animal Centre of Shenzhen University. For tumor inoculation, 2 × 10^6^ 4T1 cells suspended in 100 μL of serum-free medium were injected subcutaneously into the right hind leg of the mice. Mice can receive subsequent experiments when their tumor volume reaches approximately 100 mm^3^.

**Hemolysis assay.** Mouse red blood cell membranes (RBCs) were isolated by centrifugation (4500 rpm, 5 min) of a mixture containing blood samples (0.5 mL) and PBS (1 mL pH 7.4). After five washes with PBS, the RBCs were diluted to 5 mL with PBS. 0.3 mL of the diluted RBCs suspension was then added to PBS containing different HPPH concentrations (1.2 mL, 0 - 400 μg/mL). The mixture was vortexed and allowed to stand at room temperature for 3 h before centrifugation, and the absorbance of the supernatant at 541 nm was measured by a UV spectrophotometer. The deionized water and PBS-treated groups were used as positive and negative controls, respectively.

**Supplementary Figures:**


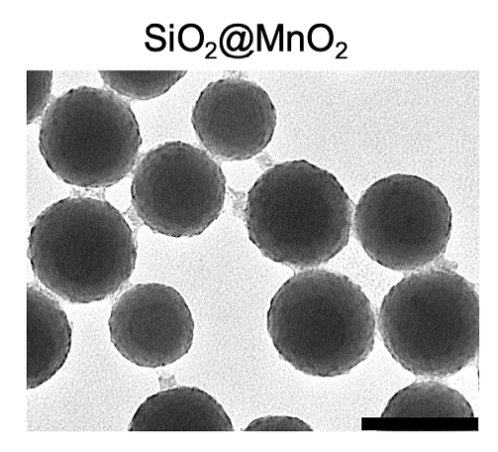


**Figure S1**. TEM image of SiO_2_@MnO_2_ nanoparticles. Scale bar: 200 nm.


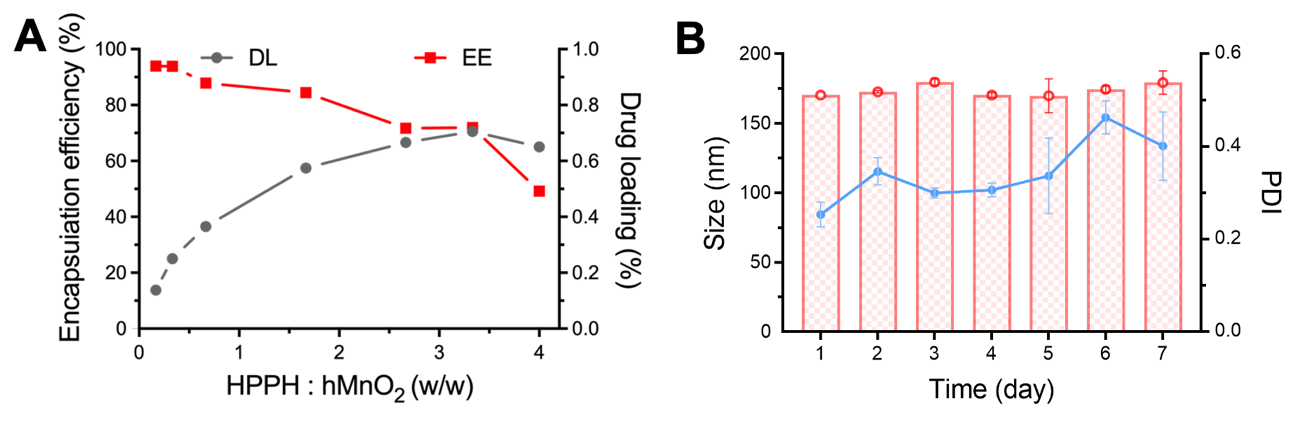


**Figure S2.** (A) Encapsulation efficiency (EE) of HPPH at various mass ratios in the hMnO_2_ nanoparticle. (B) The size and PDI changes of HL@hMnO_2_-LOx@HA stored at 4 ºC.


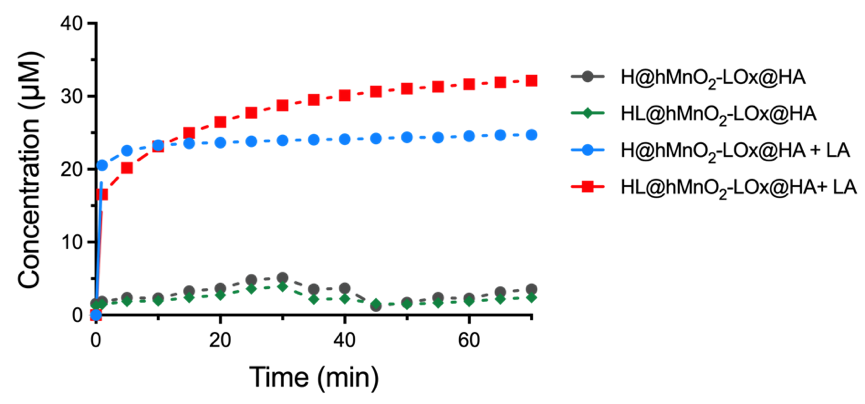


**Figure S3**. The concentrations of H_2_O_2_ generated from the reaction between LA and H@hMnO_2_-LOx@HA or HL@hMnO_2_-LOx@HA, respectively.


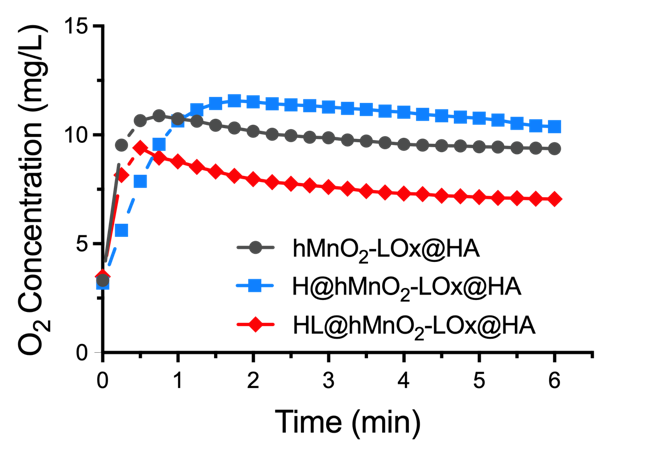


**Figure S4**. The concentrations of O_2_ during the reaction between H_2_O_2_ and indicated solutions in the degassed water ([hMnO_2_]: 10 μg/mL, [LOx]: 35 μg/mL, [H_2_O_2_]: 100 µM).


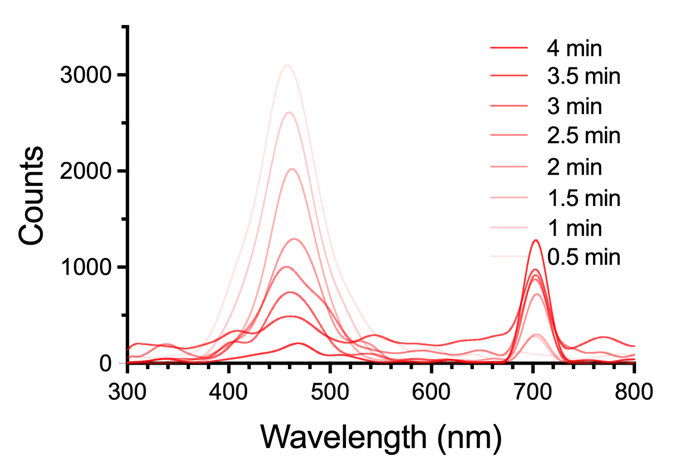


**Figure S5**. Time-resolved luminescent spectra of HL@hMnO_2_-LOx@HA in the presence of 100 µM H_2_O_2_.


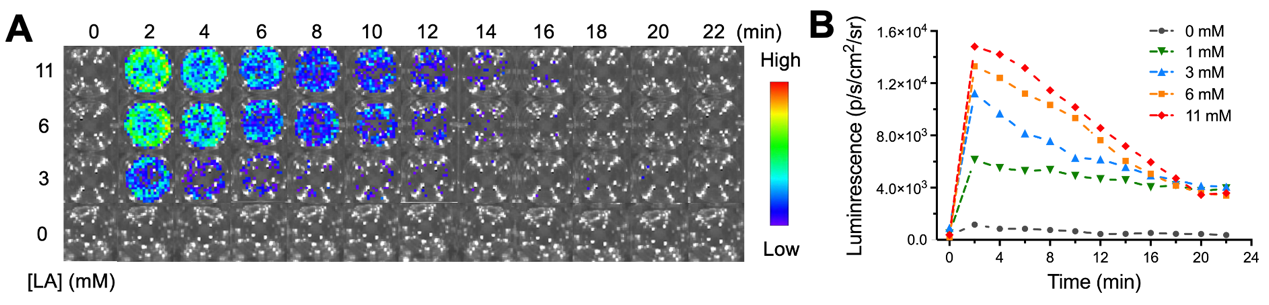


**Figure S6.** (A) Luminescence of HL@hMnO_2_-LOx@HA at various concentrations in the presence of 10 mM LA (hMnO_2_: 60 μg/mL). (B) Corresponding luminescence signal intensities during the 22-min reaction between HL@hMnO_2_-LOx@HA and LA.


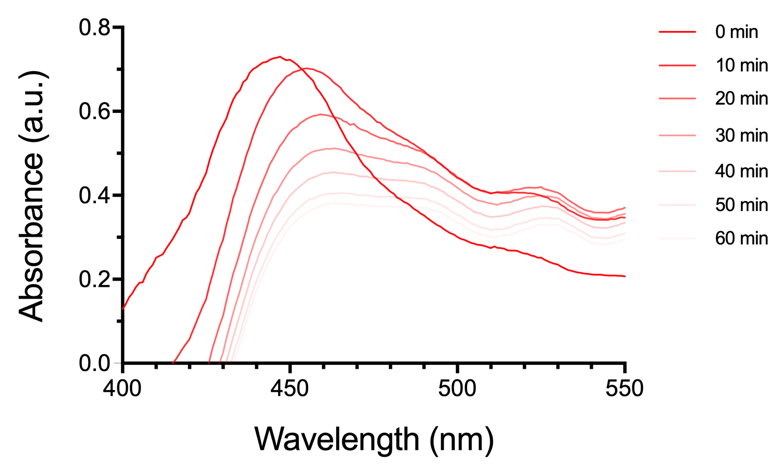


**Figure S7**. UV-vis-NIR spectra of DPBF that were used to probe the ^1^O_2_ generated from the reaction between LA and indicated solutions (hMnO_2_: 10 μg/mL, Luminol: 10 μg/mL, LOx: 35 µg/mL).


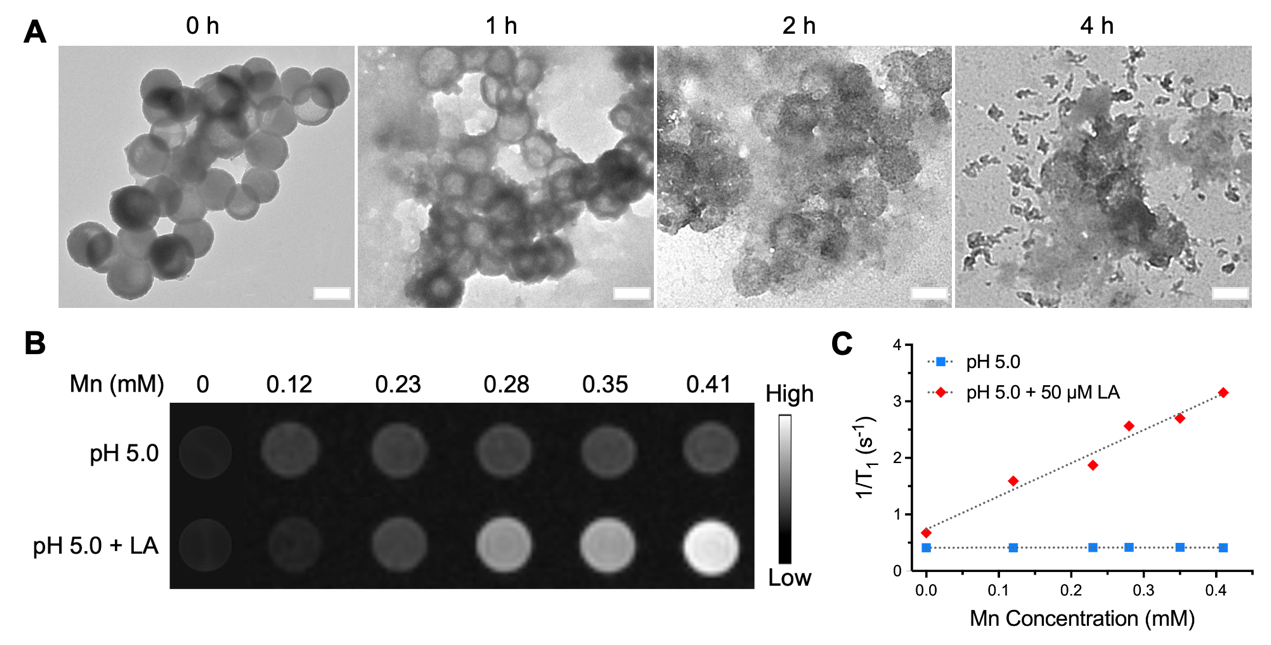


**Figure S8**. (A) TEM images of HL@hMnO_2_-LOx@HA incubated in 10 mM LA for 0, 1, 2, and 4 h. Scale bars: 200 nm. (B) *T*_1_-weighted MRI images of HL@hMnO_2_-LOx@HA at different concentrations in indicated phosphate buffer solution (PBS) solutions. (C) Corresponding 1/*T*_1_ versus Mn concentration curves.


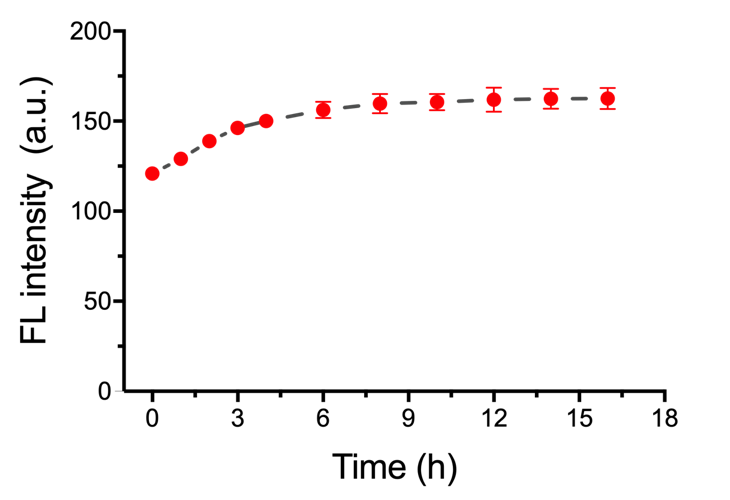


**Figure S9**. Semiquantifications of the FL intensity of 4T1 cells incubated with HL@hMnO_2_-LOx@HA for 0, 1, 4, 8, 12, and 16 h, respectively.


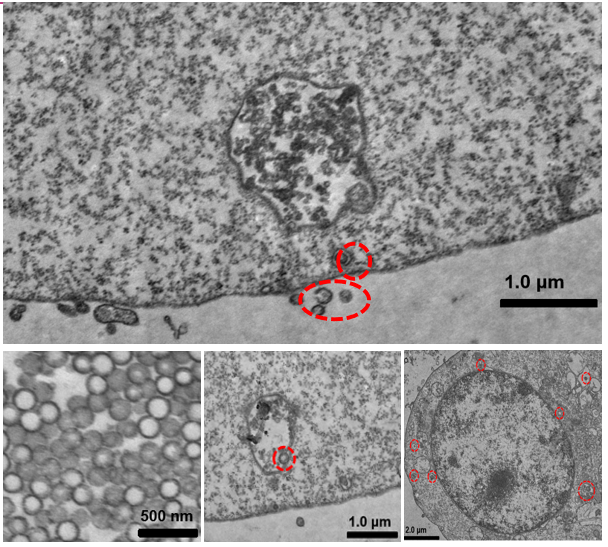


**Figure S10**. The bioTEM images of 4T1 cells incubated with HL@hMnO_2_-LOx@HA for 2 h.


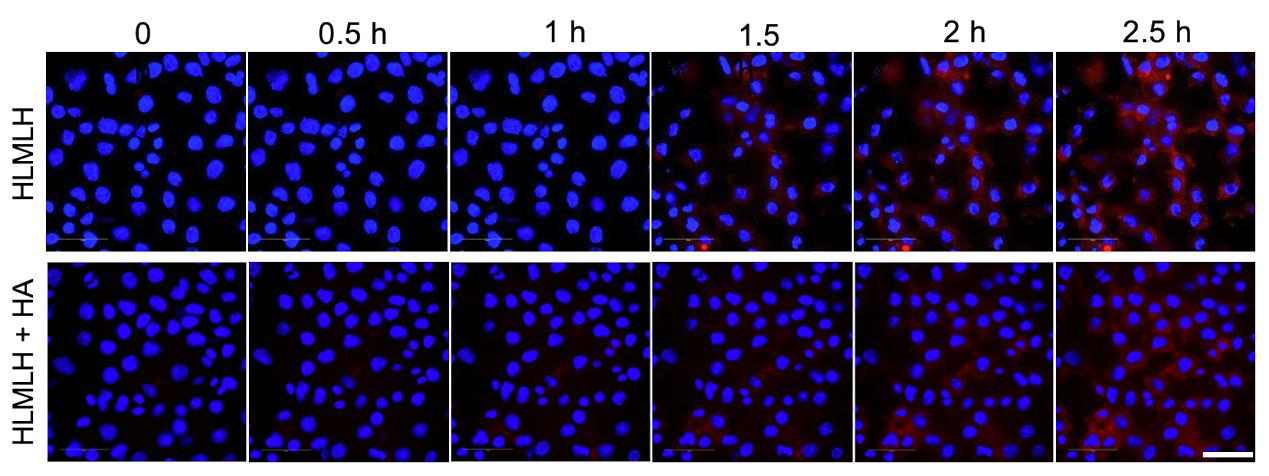


**Figure S11**. FL images of 4T1 cells incubated with HL@hMnO_2_-LOx@HA or HL@hMnO_2_-LOx@HA plus HA. Scale bars: 50 µm.


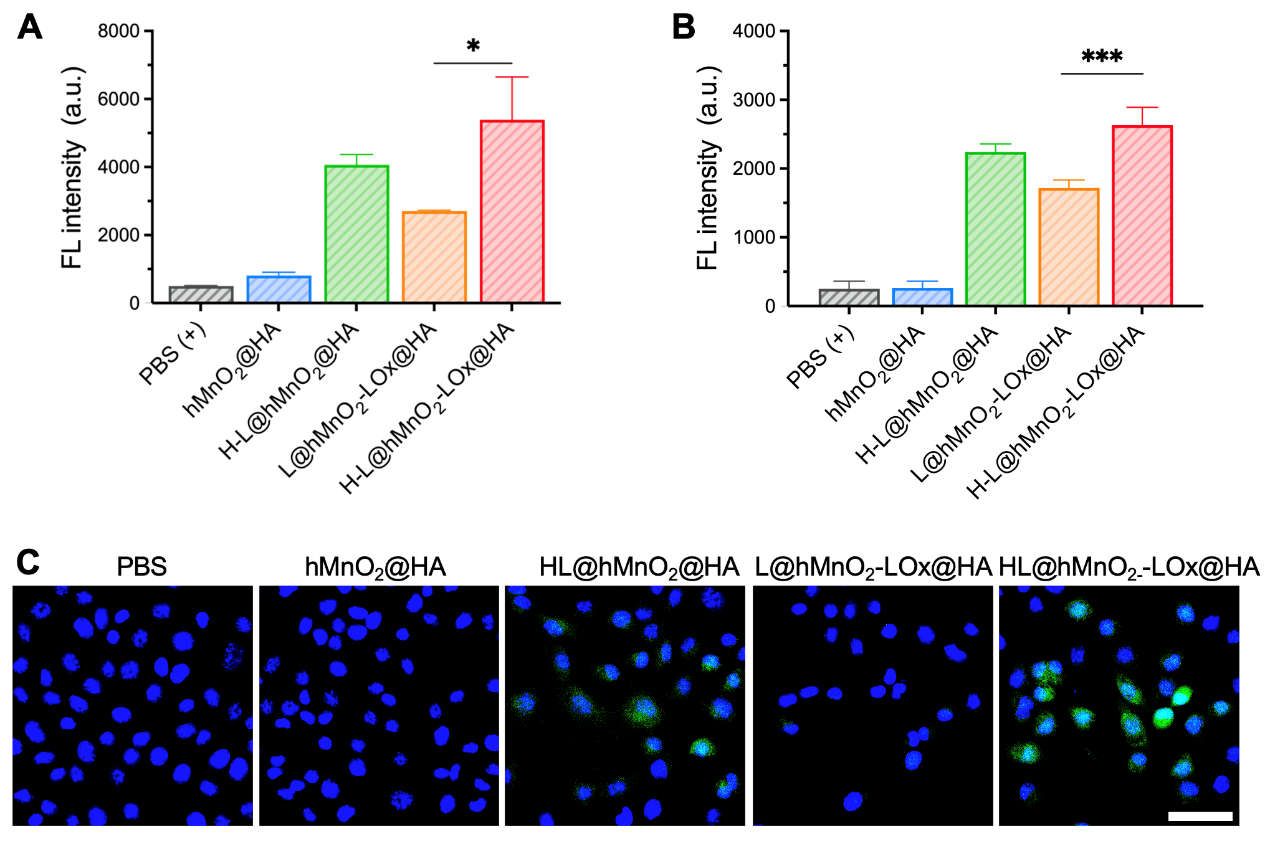


**Figure S12**. FL semi-quantification of the fluorescence of a ROS probe H_2_DCFDA in 4T1 cells after different treatments (A) under hypoxic conditions, or (B) under normoxic conditions. (C) ROS generation from 4T1 cells after different treatments under normoxic conditions. Scale bar: 50 µm.


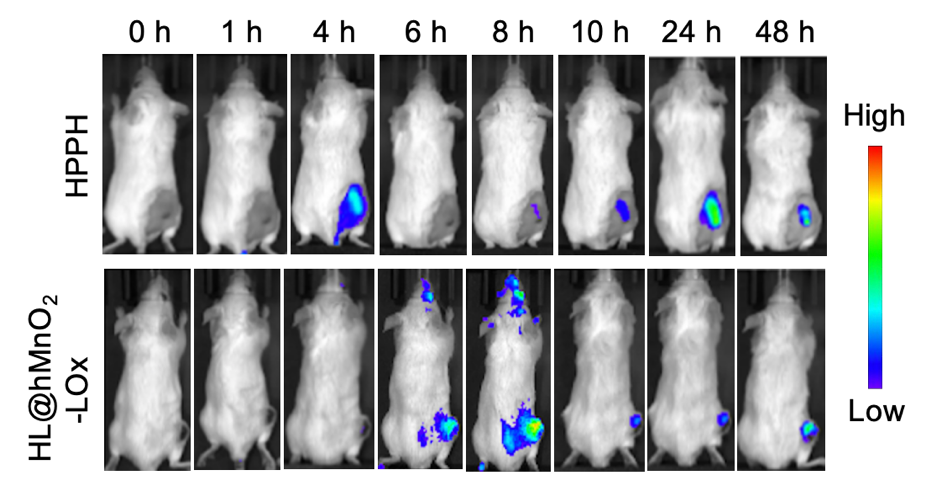


**Figure S13.** *In vivo* FL images of subcutaneous 4T1 tumor-bearing mice 0, 1, 4, 6, 8, 10, 24, 48 h post *i.v.* injection of HPPH and HL@hMnO_2_-LOx. HPPH: 10 mg/kg.


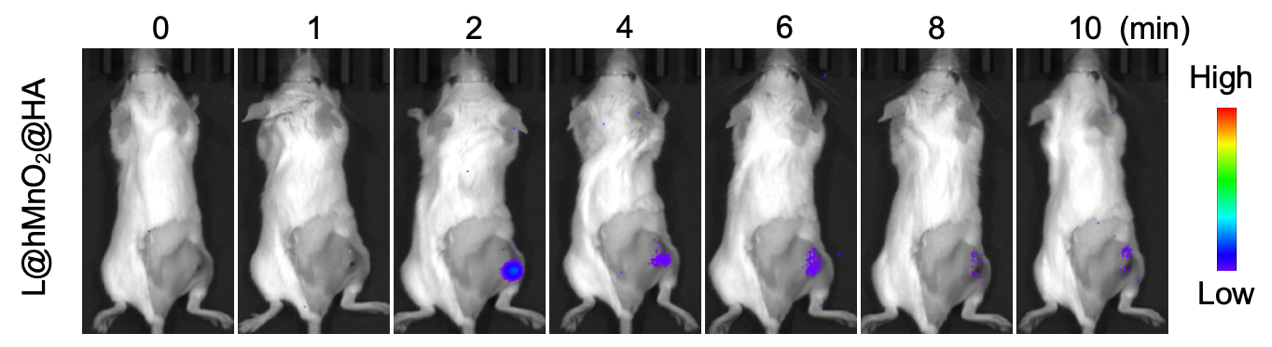


**Figure S14.** *In vivo* bioluminescent imaging of subcutaneous 4T1 tumor-bearing mice 0, 1, 2, 4, 6, 8, 10 min post *i.t.* injection of HL@hMnO_2_@HA. HPPH: 10 mg/kg.


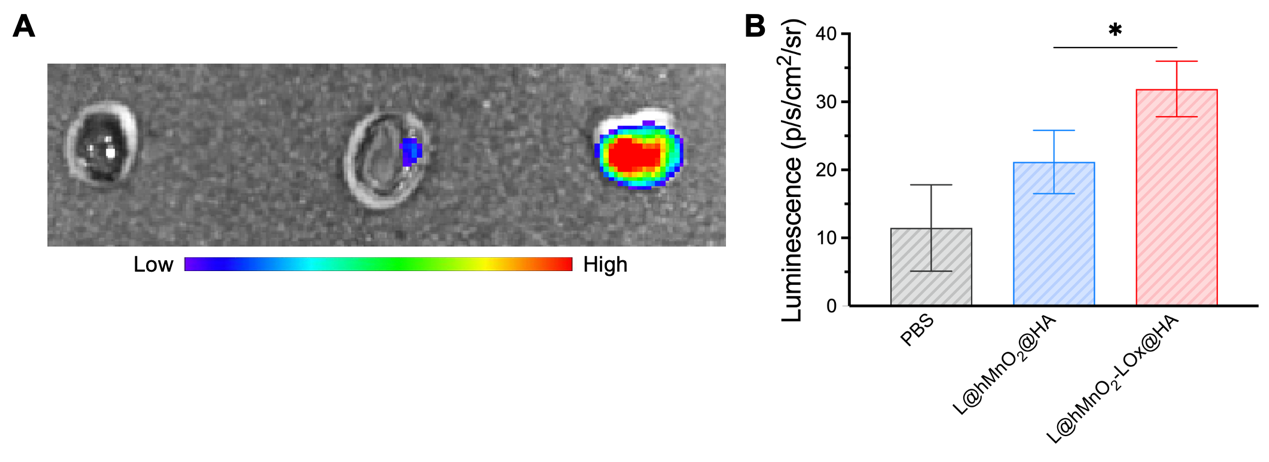


**Figure S15**. (A) *Ex vivo* bioluminescent imaging and (B) corresponding semiquantitative analysis of 4T1 tumors collected from mice 60 min post *i.t.* injection of PBS, L@hMnO_2_@HA and L@hMnO_2_-LOx@HA (from left to right).


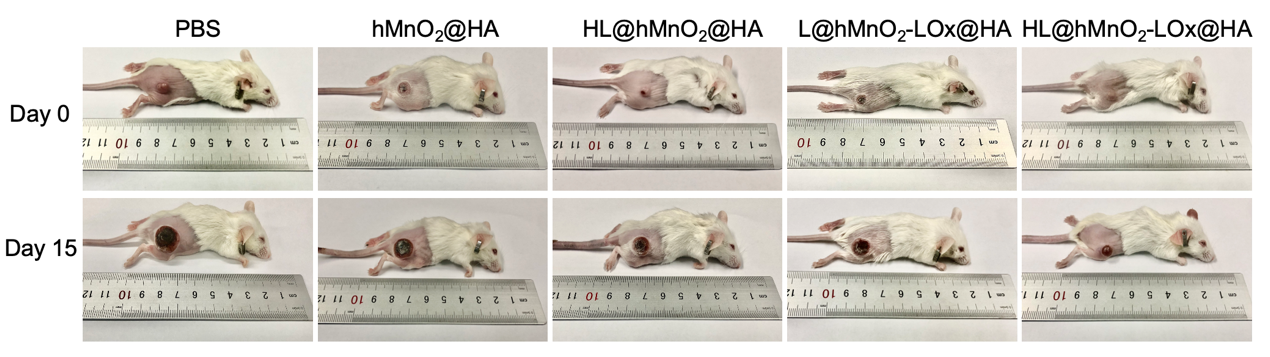


**Figure S16**. Photographs of 4T1 tumor-bearing mice 0 day and 15 days after receiving indicated treatments, respectively.


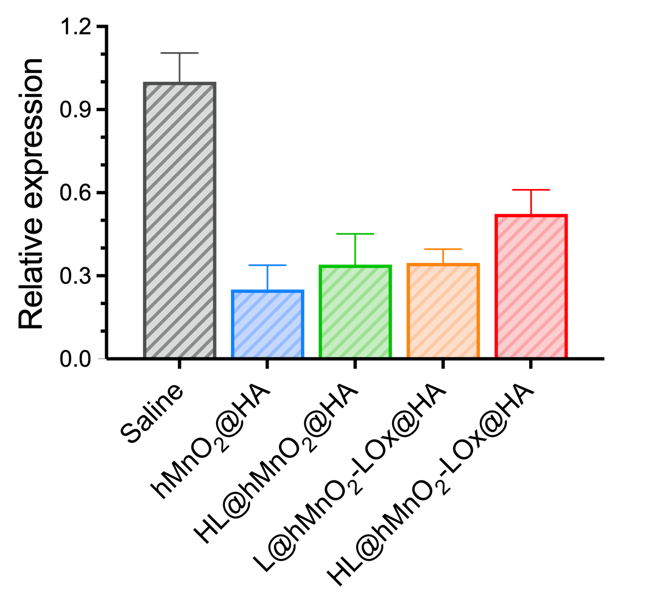


**Figure S17.** Quantification of the HIF-1α expression of tumor sections collected 15 days after indicated treatments.


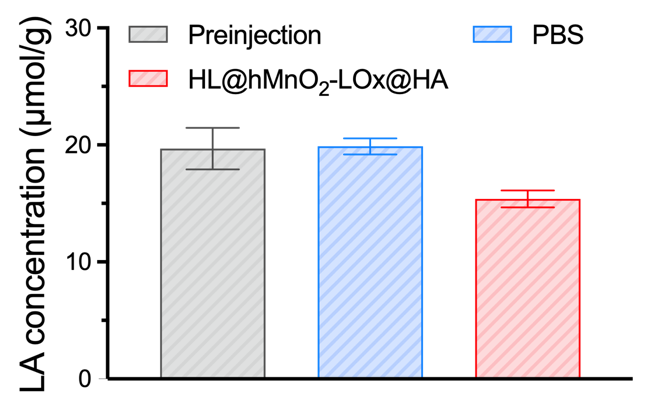


**Figure S18**. Intratumoral LA concentrations before and 8 h after *i.v.* injection of PBS or HL@hMnO_2_-LOx@HA.


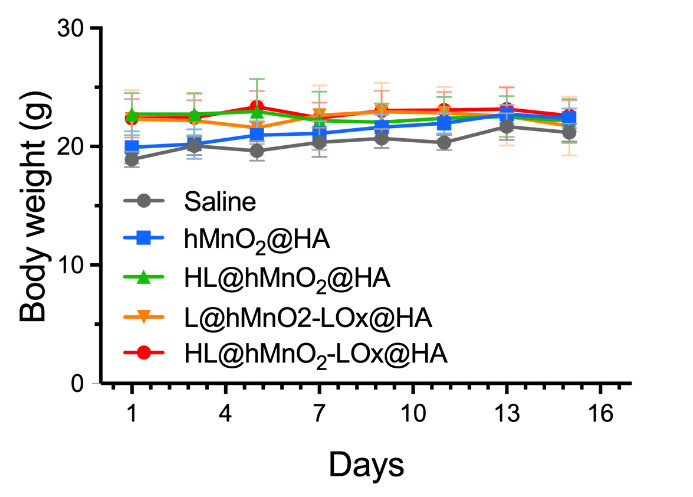


**Figure S19**. Body weight changes of 4T1 tumor-bearing mice receiving indicated treatments in the in vivo PDT experiment.


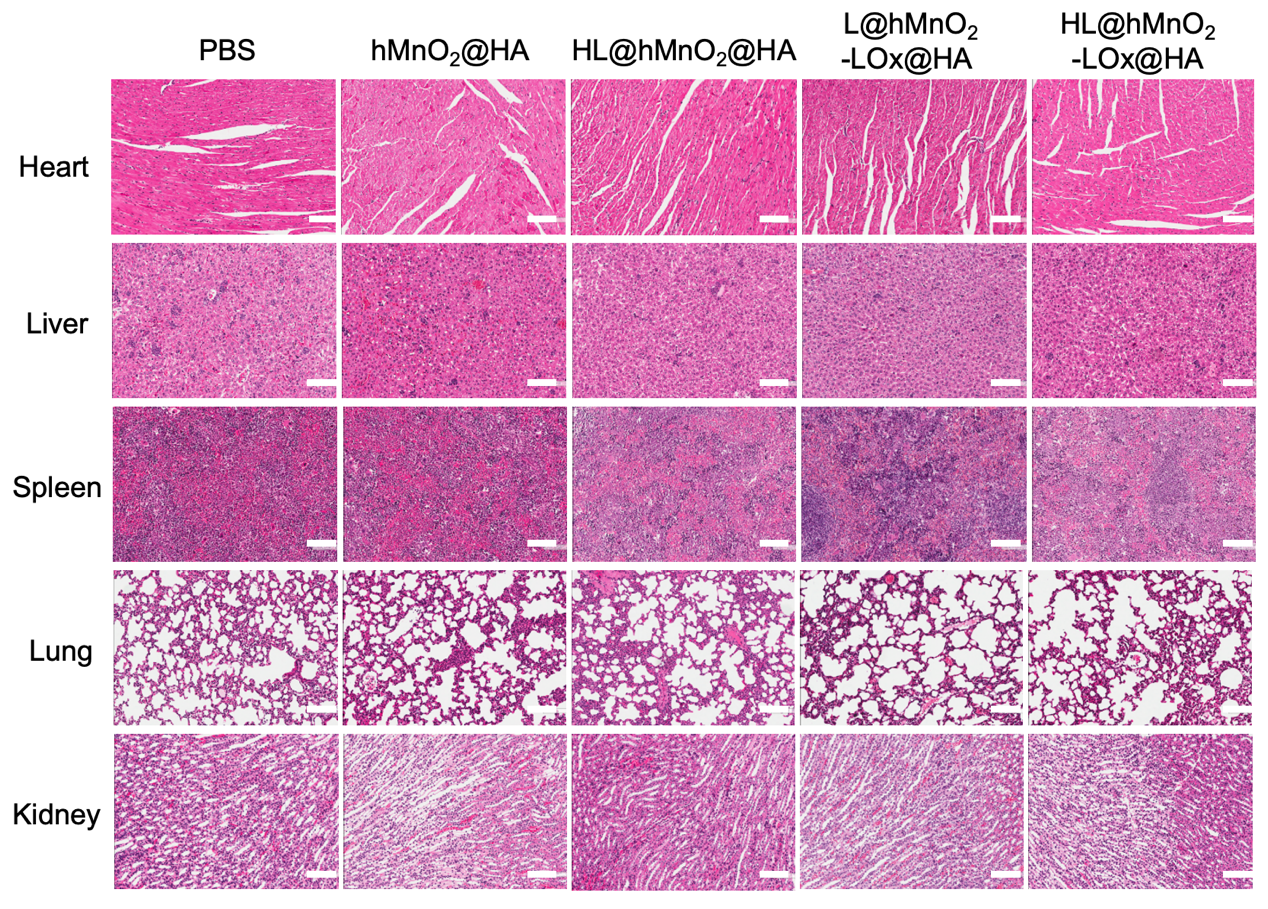


**Figure S20**. Representative H&E staining images for cellular morphology from major organs of mice at day 15 after indicated treatments. Scale bars: 300 µm.


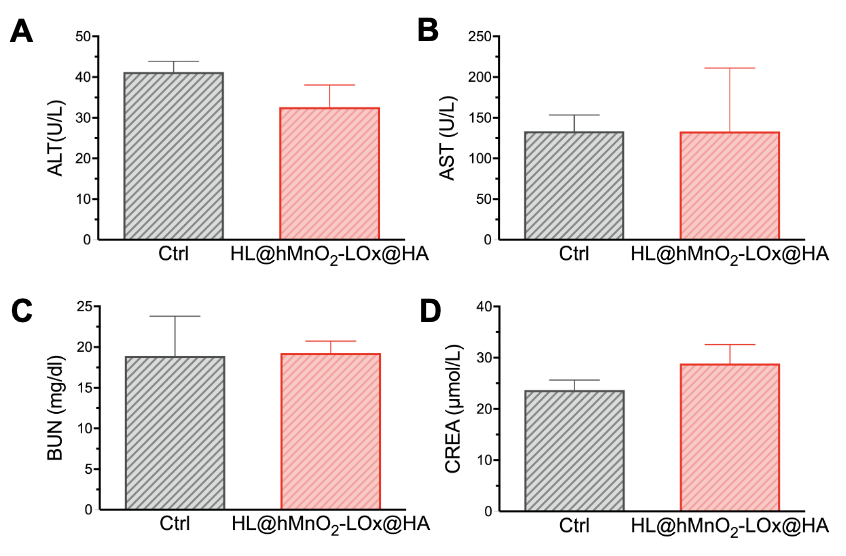


**Figure S21.** (A) Glutamic-pyruvic transaminase (ALT), (B) glutamic oxalacetic transaminase (AST), (C) urea nitrogen (BUN), and (D) creatinine (CREA) concentrations as indicators for liver and kidney functions of healthy mice before (Ctrl group) and 15 days after *i.v.* injections of HL@hMnO_2_-LOx@HA.


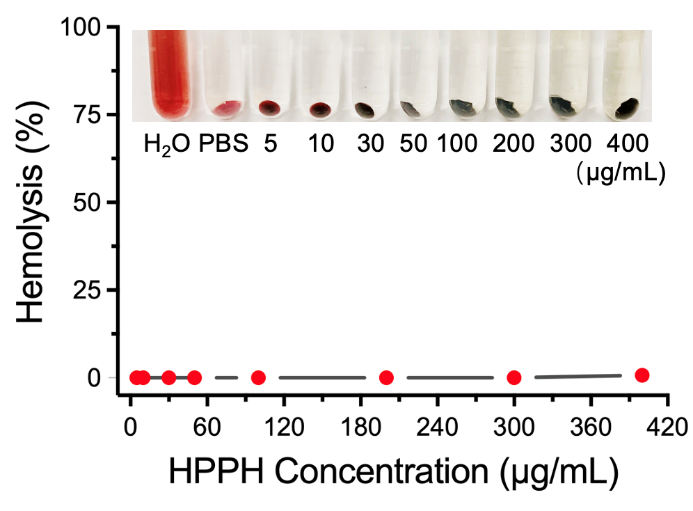


**Figure S22.** Hemolytic activity of HL@hMnO_2_-LOx@HA in the blood of healthy mice.
